# Does initial 45Gy of pelvic intensity-modulated radiotherapy reduce late complications in patients with locally advanced cervical cancer? A cohort control study using definitive chemoradiotherapy with high-dose rate brachytherapy

**DOI:** 10.2478/raon-2013-0011

**Published:** 2013-05-21

**Authors:** Shang-Wen Chen, Ji-An Liang, Yao-Ching Hung, Lian-Shung Yeh, Wei-Chun Chang, Wu-Chou Lin, Chun-Ru Chien

**Affiliations:** 1 Department of Radiation Therapy and Oncology, China Medical University Hospital, Taichung, Taiwan; 2 Department of Obstetrics and Gynecology, China Medical University Hospital, Taichung, Taiwan; 3 College of Medicine, China Medical University, Taichung, Taiwan; 4 College of Medicine, Taipei Medical University, Taipei, Taiwan

**Keywords:** cervical cancer, IMRT, brachytherapy, complication

## Abstract

**Background:**

Comparing initial 45 Gy of pelvic intensity-modulated radiation therapy (IMRT) and non-IMRT in terms of the late toxicities associated with advanced cervical cancer that has also been treated with definitive concurrent chemoradiotherapy and high-dose rate intracavitary brachytherapy (HDRICB).

**Patients and methods:**

This retrospective study included 320 stage IB2-IIIB cervical cancer patients treated with CCRT (83 IMRT and 237 non-IMRT). The two groups had similar stage and HDRICB ratings. Following 45 Gy to the pelvis, HDRICB of 24 Gy in four courses was prescribed. Late toxicities, including rectal complications (RC), bladder complications (BC) and non-rectal intestinal injury (NRRII), were scored by the Common Terminology Criteria for Adverse Events. A logistic regression was used to estimate the odds ratio (OR) of the complications.

**Results:**

With a median follow-up duration of 33 and 77 months for IMRT and non-IMRT, 33 patients had Grade 2 or higher late RC (7.2% IMRT, 11.4% non-IMRT), whereas that for BC was 40 (9.6% IMRT, 13.5% non-IMRT) and for NRRII was 48 (12.0% IMRT, 16.0% non-IMRT). The cumulative rate for total grade 3 or higher gastrointestinal or genitourinary toxicities was 8.4% and 11.8% (*p* = 0.33). IMRT did not reduce the OR for all endpoints; however, the ORs for rectum and bladder reference doses to Point A were associated with RC and BC.

**Conclusions:**

Locally advanced cervical cancer patients treated with initial 45Gy of pelvic IMRT and HDRICB have similar treatment-related late toxicities as those treated with non-IMRT. Optimization of the brachytherapy scheme is essential to minimize late toxicities.

## Introduction

The use of intensity-modulated radiation therapy (IMRT) for gynecological malignancies has grown considerably^1^, despite limited data on long-term toxicities and survival. Several studies have shown that IMRT reduces bowel, rectal, bladder, and bone marrow dose and is associated with lower rates of early gastrointestinal, genitourinary, and hematological toxicity compared with conventional techniques.^1^–^9^ Furthermore, many IMRT studies have been associated with better outcomes.^1^–^3^ Although current findings suggest a favorable impact of IMRT on outcomes, so far, no randomized phase 3 trial has been conducted to examine its benefits probably due to ethical concerns. Despite IMRT became increasingly popular for gynecological cancer patients treated at many institutions, it remains unclear whether the lower rates of toxicity observed in our patients with an intact uterus. In this circumstance, brachytherapy might be an important factor affecting local control and late toxicities.^10^ Particularly, concurrent cisplatin may also cause an increased incidence of hematological and gastrointestinal side effects.^11^ A large cohort study has compared the impact on patients of IMRT with patients treated by non-IMRT as a control cohort.^2^ They showed that pseudo–step-wedge intensity modulation can achieve better survival and less treatment-related late toxicities. However, currently this technique is not commonly used in most institutes. Furthermore, all the patient and treatment variables should be compared together before IMRT can be assumed to improve therapeutic ratio.

Previous studies have suggested that organ motion and deformation of the target volumes occur during IMRT for cervical cancer.^12^–^16^ Furthermore, a study showed a relative reduction in volume during treatment of 0.02–0.79.^16^ In consequence, adjacent normal organs might receive an unexpected irradiation dose following the shrinkage of the tumor during fractionated external beam radiotherapy (EBRT). The combination of unpredictable organ motion and substantial tumor regression has resulted in a consensus guideline suggestion that margins of 1.5 to 2 cm around clinical target volume (CTV) are to be recommended if good quality daily soft tissue verification was available during treatment.^17^ Thus, certain limitations exist when the physician try to reduce the physical dose affecting the adjacent normal tissues when using IMRT.

In definitive chemoradiotherapy for locally advanced cervical cancer, in which brachytherapy is still an essential component of radiotherapy, the net impact of pelvic IMRT on late toxicities remains to be determined. We conducted a retrospective cohort comparison study with controlled stage and demographic distributions in order to clarify the impact of IMRT on late complications. All the included patients were treated with the same pelvic EBRT dose of 45 Gy in 1.8 Gy daily fractions, concurrent chemotherapy and a standard brachytherapy scheme, namely high-dose rate intracavitary brachytherapy (HDRICB). Furthermore, several patient and treatment variables were analyzed to assess the impact of IMRT. The result will be helpful to institutions where the cost-effectiveness of IMRT is a concern, particularly where resources are limited.

## Patients and Methods

### Patients

This retrospective cohort study included 320 patients with stage IB2-IIIB cervical cancer treated with curative intent (83 IMRT and 237 non-IMRT) between 2002 and 2009 at China Medical University Hospital. Since 2007, most patients with newly diagnosed cervical cancer have been prospectively treated with an IMRT to the pelvis, and are labeled as the IMRT group. All patients in the two groups received comprehensive pretreatment workup, including computed tomography, and completed an allocated CCRT course. Positron emission tomography (PET) was used for the workup in selected patients who were observed to have a maximum diameter of lymph node of more than 1 cm. The two groups had similar stage and histological distributions. Patients with positive paraaortic lymph node, an extended field, or distant metastasis at diagnosis were excluded. The characteristics of the two groups are listed in Table 1. Except for the follow-up duration, all the patient-related or brachytherapy-related factors were similar. The study was approved by the institutional review board.

### Conventional external beam radiotherapy

All patients underwent CT-based planning with custom immobilization. Initially, the whole pelvis was treated with 10 MV X-ray via anterior and posterior parallel fields or box fields when the AP diameter was greater than 18 cm. We prescribed an EBRT dose of 45 Gy in 25 fractions over 5 weeks to the whole pelvis. Generally, a field margin of at least 1.5 cm around the gross tumor was used, as described previously.^18^ Then, bilateral parametrial disease was boosted to 50.4 to 59.6 Gy via anterior and posterior parallel field technique with a rectangular central shielding of 4 cm width. By this technique, the mean doses to the rectum were kept less than 10% of the boost doses, whereas that for the bladder fewer than 40%. Accordingly, we made the assumption that the major contributor of EBRT to the both organs would derive from the initial pelvic radiotherapy (RT).

### IMRT technique

Intensity-modulated radiation therapy plans consisted of 7 coplanar fields using 10 MV photons. The prescription dose to the whole pelvis was 45 Gy. Following IMRT, bilateral parametrial disease was boosted to 50.4 to 59.6 Gy via anterior and posterior parallel fields with the same central shielding as mentioned above.

In the IMRT group, the CTV included the gross disease, cervix, parametrium, uterus, superior half of the vagina, cardinal ligament, presacral region, and regional lymph nodes (common, internal, and external iliac). Inguinal nodes were treated in women with involvement of the inferior third of the vagina. The CTV delineation was similar to the consensus guidelines on CTV delineation emerged.^17^ Uniform planning margins were added to account for organ motion and setup uncertainty. According to previous studies^12^,^13^, we applied a 15 mm planning margin around the cervix, a 10 mm margin around the uterus and the vagina, and a 8 mm margin around the remainder of the CTV.

**FIGURE 1:**
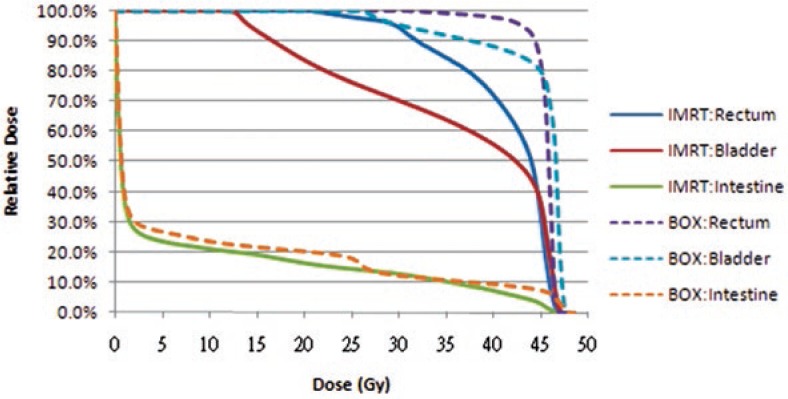
Dose-volume histiogram of adjacent normal organs between the IMRT and 4-field box field for one patient.

Target planning constraints became standardized as follows: (1) more than 97% of the planning target volume (PTV) receives more than 97% of the prescription dose, (2) less than 1% of the PTV receives less than 93% of the prescription dose, (3) less than 5% of the PTV receives more than 107% of the prescription dose. When prescribing 45 Gy of dose to the whole pelvis, normal tissue planning constraints were consistent and were as follows: (1) rectum, less than 50% of volume receives greater than 45Gy; (2) bladder, less than 50% of volume receives greater than 45Gy, and (3) non-rectal bowel, less than 10% of volume receives greater than 45 Gy. No special constraint was used for the bone marrow. The constraints on dose-volume histogram (DVH) for a normal organ given above were not mandatory when the physician considered it necessary not to compromise the PTV coverage. Figure 1 depicts the DVH of adjacent normal organs between the IMRT and box field for one patient.

### Brachytherapy

After adequate tumor regression, HDRICB was performed using an Ir-192 remote after-loading technique at 1 week intervals and concurrently with pelvic irradiation or parametrial boosting. The standard prescribed dose for each HDRICB was 6.0 Gy to Point A for four sessions. The Point A dose was reduced to 5.0 Gy for those with higher reference doses to the rectum or bladder, or whose age was over 70 years. The total prescribed Point A doses (EBRT + HDRICB) of a radiobiological equivalent dose in 2 Gy fractions (EQD_2_) ranged from 69.25 to 84.25 (median, 76.25). The details of the radiotherapy technique have been reported previously.^19^

The geometric sparing factor (GSF) is defined as the average of the ratios between the reference dose and the Point A dose during each HDRICB insertion. The mean GSF for the rectum (abbreviated as RGSF) = the average of the ICRU rectal dose/Point A dose. The mean GSF for the bladder (abbreviated as BGSF) = the average of the ICRU bladder dose/Point A dose. The description of GSF was described in our previous study.^19^ Accordingly, in this study the RGSFs and BGSFs were stratified with cuff-offs of 0.7 and 0.9, respectively.

### Chemotherapy

Chemotherapy consisted of cisplatin delivered weekly at a dose of 40 mg/m^2^ intravenously, with a total maximal dose of up to 60 mg. The first cycle of cisplatin was initiated at the first RT treatment. In accordance with the duration of RT, the treatment plan included a total of five to six cycles of cisplatin. The detailed drug administration protocol was described in our previous study.^18^

### Follow-up

After completion of radiotherapy, patients received regular follow-up every 1 to 2 months for the first year, and then every 3 months afterward. A pelvic examination was performed during each follow-up; in addition, tumor markers (squamous cell carcinoma antigen and carcinoembryonic antigen) were checked. A radiographic examination was carried out every 3 to 6 months and routine urine and stool examinations were done every 6 to 12 months. Patients who had persistent cramping abdominal pain, bloody stools or hematuria underwent sigmoidoscopy or cystoscopy to identify the source of bleeding, and underwent blood counts every 2 to 4 weeks for surveillance of the severity of the complications.

### Complication analysis

Common Terminology Criteria for Adverse Events Version 3.0 was used to score the maximum late toxicities, including rectal complications (RC), bladder complications (BC), non-rectal intestinal injury (NRRII), and leg edema. The definition of the NRRII was reported in our previous study.^20^ There were several study endpoints, including grade 2 and higher RC, grade 2 and higher BC, grade 2 and higher NRRII, total grade 2 and higher gastrointestinal or genitourinary complications, and total grade 3 and higher gastrointestinal or genitourinary complications.

### Statistics

A comparison of the categorical variables was performed using the χ^2^ test. A Student’s *t* test was used to compare differences in continuous variables when patients were stratified into the two groups. A logistic regression was used to estimate the odds ratio (OR) of complications among the variables examined. Although we believe that a longer follow-up duration is needed to estimate survival differences between the two groups, cause-specific survival (CSS) and disease-free survival (DFS) were calculated using the Kaplan-Meier method to provide preliminary results for the two treatment regimes. Statistical significance was considered to have occurred when a two-sided *p* value of <0.05 was found. Patient survival was measured from the date of radiotherapy initiation to the last follow-up. The latency of complications was measured from the end of radiotherapy to the last follow-up. All statistical analyses were performed using a commercial software package (SPSS 13.0 for Windows, Chicago, IL, USA).

## Results

The mean follow-up duration for the 320 patients enrolled at the time of last visit was 62 months (33 months IMRT, 77 months non-IMRT). At the time of last follow-up, 78 patients died of cancer, 10 in the IMRT group, and 68 in the non-IMRT group. Eighty-eight patients had developed a recurrence, 16 in the IMRT group, and 72 in the non-IMRT group. There was a similar pattern of recurrences between the two groups, with more than 80% of patients having distant recurrences. The 3 years CSS for the IMRT and non-IMRT groups were 86% and 76% (*p* = 0.095), whereas the 3 years DFS was 78% and 74% (*p* = 0.37) (Figure 1).

Of the patients, 33 patients had Grade 2 or higher late RC (6 IMRT, 27 non-IMRT). In all, 40 patients had Grade 2 or higher late BC (8 IMRT, 32 non-IMRT), whereas 48 patients had Grade 2 or higher NRRII (10 IMRT, 38 non-IMRT). The median time for the development of RC, BC and NRRII was 12 months (range, 7–35 months), 19 months (range, 3–49 months) and 13 months (range, 3–28 months). The cumulative rate for total grade 2 or higher gastrointestinal or genitourinary complications was 22.9% (19/83) in the IMRT group and 30.0% (71/237) in the non-IMRT group (*p* = 0.24), whereas that for total grade 3 or higher complications was 8.4% (7/83) among IMRT patients and 11.8% (28/237) among non-IMRT patients (*p* = 0.33). Among IMRT patients, the cumulative rate for grade 3 or higher RC, BC and NRRII were 2.4%, 3.6% and 4.8%, respectively. Details of the various complication endpoints for the IMRT and non-IMRT patients are listed in Table 2.

The correlation of patient and treatment related factors with several complication endpoints are summarized in Tables 3, 4 and 5. Logistic regression analysis demonstrated a high risk of Grade 2 and higher RC in patients who developed BC complications (*p* = 0.000; odd ratio [OR], 2.18, 95% confidence interval [CI], 1.44–3.30) and in those with higher RGSF values (*p* = 0.003; OR, 3.36; 95% CI, 1.52–7.43). Furthermore, there was a high risk of Grade 2 or higher BC in patients who developed RC (*p* = 0.000; OR, 2.47, 95% CI, 1.65–3.70) and in those with a higher BGSF values (*p* = 0.04; OR, 2.01; 95% CI, 1.01–4.32). There were high risk factors for Grade 2 or higher NRIII among those patients having ≥grade 2 RC (*p* = 0.001; OR, 1.99; 95% CI, 1.26–3.16). In general, IMRT was associated with a lower risk of developing most late toxicities; however, the trend was not statistically significant. Age, clinical stage, diabetes, parametrial dose and ICB number were not associated with increased risk of late sequelae by multivariate analysis.

To minimize the confounding impact of the GSFs on toxicities analysis, patients were further stratified according to lower and higher 50% percentile of the GSFs for rectum and bladder. As showed in Table 6, there was no significant difference between IMRT and non-IMRT groups for developing grade 2 or greater complications when they were categorized according to the median GSF values.

## Discussions

The application of IMRT is believed to result in the organs at risk being exposed to a lower dose of radiation and consequently there will be a reduction in toxicities. Currently, long-term comparison data remain limited in the setting of definitive CCRT for locally advanced cervical cancer. Taking into consideration that the follow-up duration in this study was not very long (median, 33 months), we found that initial 45 Gy of IMRT to the pelvis did not reduce significantly the long-term complications as reported in other comparable studies.^2^,^3^ By analyzing several complication endpoints, not simply classify the late toxicities as gastrointestinal and genitourinary system, or solely reporting grade 3 late toxicities, this study comprehensively explored the CCRT-related toxicities during definitive treatment for cervical cancer. Unlike those treated in the postoperative setting, in which irradiation dose is mainly prescribed by external beam, brachytherapy always plays an important role in determining the final outcome for patients with an intact uterus. Thus, the application of IMRT ought to be one of the determinants of outcome. The impact of external beam and brachytherapy should be assessed together due to the cumulative effect of both modalities.

With the longest median follow-up duration among other similar IMRT studies^1^–^3^,^9^, we report here that the cumulative rates for grade 3 or higher RC, BC and NRRII to be 2.4%, 3.6% and 4.8%. Furthermore, our study disclosed that total grade 3 or greater toxicities among patients were 8.4%. Despite the significant variation in follow-up duration, the figure seems to be comparable with the other studies.^1^–^3^,^9^ In a study by Hasselle *et al*.^1^, the rate of grade 3 and greater complications in 89 patients with intact cervix treated with IMRT plus low-dose rate was 4% and 5% for gastrointestinal and genitourinary system, respectively. Particularly, Kidd *et al.*^2^, showed a great difference in the incidence of late toxicities between the groups. Their IMRT group had only a 6% rate of Grade 3 or greater GI or GU toxicity, versus 17% for the non-IMRT group (*p* = 0.0017), whereas the median follow-up duration for IMRT and non-IMRT was 22 and 72 months, respectively. Despite the majority of the urinary and rectal complications occurring within 2 to 3 years after the completion of therapy, the risk of developing grade 3 late complications might occur up to 25 years after treatment, as pointed out by Eifel *et al*.^21^ Thus, a long-term observational study is essential to verify the findings of the recent IMRT studies.

This study showed that RGSF and BGSF were treatment-related factors in determining grade 2 or greater RC and BC, respectively. In addition, a close relationship between the two complications was observed. All these findings verify the results of our previous study.^19^ Thus, an optimization of the HDRICB is essential to minimizing late complications. Although our current treatment scheme (45 Gy to whole pelvis plus 24 Gy of HDRICB Point A divided into four courses) is able to achieve a similar outcome compared with other IMRT studies^1^–^3^,^9^, many aspects remains to be improved. First, it is imperative to continue efforts to explore genetic predisposition in order to determine which patients are susceptible to radiation-induced normal tissues injury. Second, image-based HDRICB studies are appropriate approaches that might be used to minimize complications further.^22^ In this context, despite the fact that our study classified late gastrointestinal toxicities into RC and NRRII, the irradiated intestinal DVH due to each brachytherapy remains unknown. This is also a major drawback when investigating the NRRII. In the future, image-based brachytherapy might be used to calculate the intestinal volume during each application. By counting the intestinal DVH due to IMRT and brachytherapy separately and together, a comprehensive dosimetric analysis for NRRII could be obtained.

The results should be interpreted with several limitations. When comparing outcomes using a historical control, the follow-up duration between groups is always a weakness when presuming the final treatment outcome. Although IMRT patients had a shorter follow-up duration, the result showed a trend towards similar complications between the groups. Accordingly, we assumed the irradiated strategy of combining IMRT with HDRICB needs to be optimized further. Second, despite most volumes of the rectum and bladder were spared during the parametrial boost field, the IMRT benefit could be somewhat diluted by the non-IMRT boost technique. To optimize the preferable dose distribution made by IMRT, our study highlighted a need to develop a special IMRT boost technique to exactly match the isodose of brachytherapy. Finally, larger margins in the pelvic IMRT tend to diminish the degree of organ sparing; the advantage of the dose distribution from IMRT should be intensified by the general implementation of image-guided RT and adaptive RT to circumvent interfraction or intrafraction motion. By correcting the uncertainty after tumor regression, the adaptive approaches allow a reduction in the margin that is added to the CTV. In this way, the therapeutic index ought to be promoted. Nonetheless, by investigating several complication endpoints via an analysis of many patient and treatment factors, our experience will be helpful to those institutions where HDRICB is performed. In the future, monitoring the information of quality of life before and after CCRT would be essential to clarify the benefit of IMRT.^23^ Although we failed to demonstrate the robust advantage of IMRT in definitive CCRT for advanced cervical cancer, the result will also help where the cost-effectiveness of IMRT is a concern, particularly when the resources are limited.

## Conclusions

Locally advanced cervical cancer patients treated with a combination of 45Gy of pelvic IMRT and HDRICB have similar treatment-related late toxicities compared with those treated with a similar non-IMRT regimen. The ratios of rectum and bladder reference doses to Point A are associated with RC and BC. For those institutions where HDRICB is performed, optimization of the combining IMRT and brachytherapy scheme is essential to minimize late toxicities.

## Figures and Tables

**FIGURE 2:**
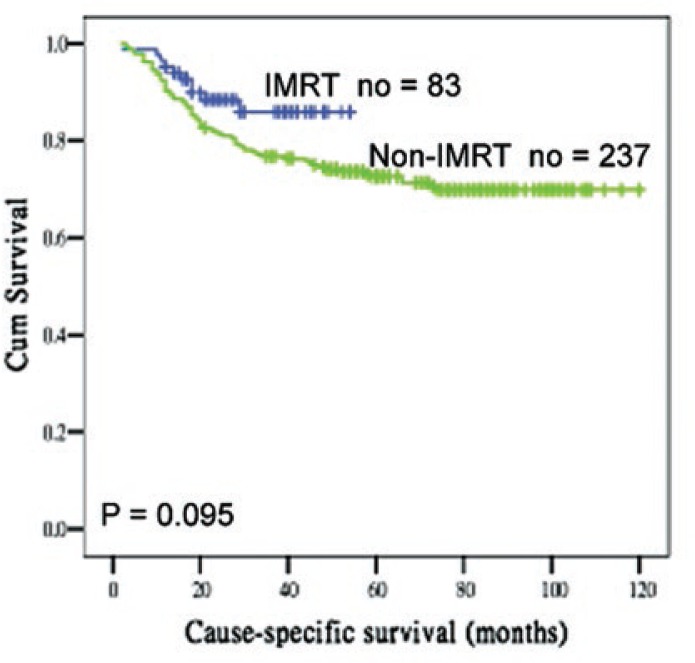
Cause-specific survival curves according to IMRT and non-IMRT groups.

**FIGURE 3:**
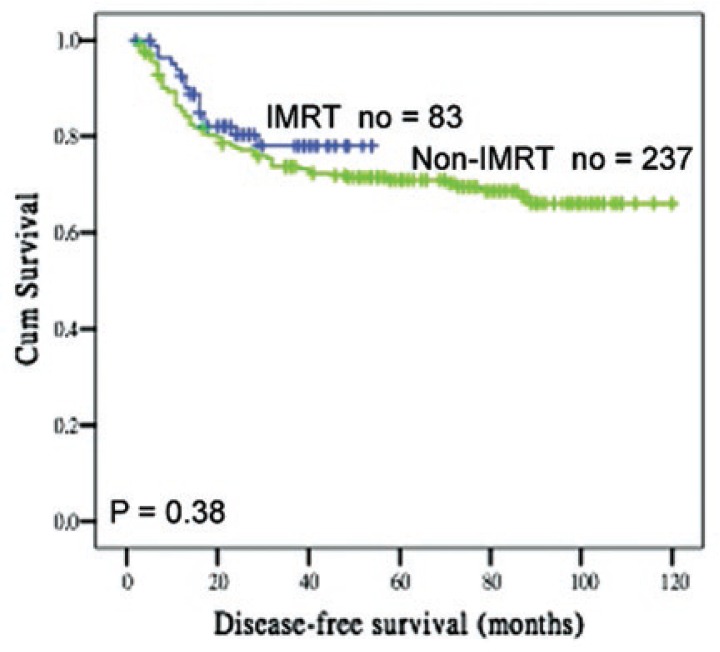
Disease-free survival curves according to IMRT and non-IMRT groups.

**TABLE 1:** Patient and tumor characteristics for the IMRT and non-IMRT groups

**Characteristicss**	**IMRT N=83**		**Non-IMRT N=237**		***p* value**

	**n**	**(%)**	**n**	**(%)**	
Median age (years)	54		54		0.47*
Stage					0.80
IB2-IIA2	20	(24.1)	49	(20.7)	
IIB	38	(45.8)	113	(47.7)	
IIIA-IIIB	25	(30.1)	75	(31.6)	
Histology					0.87
squamous cell ca.	74	(89.2)	214	(90.3)	
adenocarcinoma or adenosquamous	9	(10.8)	23	(9.7)	
Pelvic lymph node status					0.83
negative	59	(71.1)	181	(76.4)	
positive	24	(28.9)	56	(23.6)	
Patients with brachytherapy number = 5	12	(14.5)	24	(10.1)	0.75
Patients with reduced point A dose (≤5 Gy)	17	(20.5)	33	(13.9)	0.37
Six or more courses of concurrent cisplatin	58	(71.1)	164	(69.2)	0.90
Diabetes	8	(9.6)	22	(9.2)	0.88
Median follow-up (months)	33 (range:13∼54)		77(range: 36∼115)		0.000*

* examined by *t*-test

**TABLE 2:** Late complications between the IMRT and non-IMRT groups

**Classification of complication**	**IMRT N=83**		**Non-IMRT N=237**		**total**	***p* value**

	**n**	**(%)**	**n**	**(%)**		
Grade 2 or higher RC	6	(7.2)	27	(11.4)	33	0.24
Grade 3 or higher RC	2	(2.4)	6	(2.5)	8	0.99
Grade 2 or higher BC	8	(9.6)	32	(13.5)	40	0.25
Grade 3 or higher BC	3	(3.6)	14	(5.9)	17	0.32
Grade 2 or higher NRRII	10	(12.0)	38	(16.0)	48	0.38
Grade 3 or higher NRRII	4	(4.8)	16	(6.7)	21	0.76
Total grade 2 or higher gastrointestinal or genitourinary complications	19	(22.9)	71	(30.0)	90	0.24
Total grade 3 or higher gastrointestinal or genitourinary complications	7	(8.4)	28	(11.8)	35	0.33
Grade 2 or higher leg edema	9	(10.8)	16	(6.8)	25	0.43

RC = rectal complication; BC = bladder complication; NRRII = non-rectal intestinal injury.

**TABLE 3:** Multivariate logistic regression estimated odds ratios (OR) for developing grade 2 or higher RC across different variables

**Variables**	***p* value**	**OR**	**95% CI**
Age < 65 vs. ≥65 years	0.12	1.34	0.84∼2.82
Age < 70 vs. ≥70 years	0.43	1.12	0.38∼2.29
Diabetes negative vs. positive	0.70	1.32	0.39∼8.31
Stage IB2-IIA vs. IIB-IIIB	0.93	1.03	0.52∼2.07
Stage IB2-IIB vs. IIIA-IIIB	0.44	0.68	0.26∼1.77
Non-IMRT vs. IMRT	0.67	0.76	0.22∼2.56
Parametrial dose ≥54 vs. > 54 Gy	0.065	3.49	0.93∼13.17
RAL-IC number 4 vs. 5	0.40	1.64	0.51∼5.25
RGSF < 0.7 vs. ≥0.7	0.003	3.36	1.52∼7.43
≥Grade 2 BC	0.000	2.18	1.44∼3.30

RC = rectal complication; BC = bladder complication; NRRII = non-rectal intestinal injury; RGSF = geometric sparing factor of the rectum; BGSF = geometric sparing factor of the bladder.

**TABLE 4:** Multivariate logistic regression estimated odds ratios (OR) for developing grade 2 or higher BC across different variables

**Variables**	***p* value**	**OR**	**95% CI**
Age < 65 vs. ≥65 years	0.57	0.69	0.18∼2.56
Age < 70 vs. ≥70 years	0.66	1.43	0.32∼6.40
Diabetes negative vs. positive	0.47	1.61	0.43∼8.52
Stage IB2-IIA vs. IIB-IIIB	0.66	1.13	0.65∼1.97
Stage IB2-IIB vs. IIIA-IIIB	0.36	0.68	0.29∼1.57
Non-IMRT vs. IMRT	0.10	0.42	0.15∼1.19
Parametrial dose ≥ 54 vs. > 54 Gy	0.57	0.78	0.29∼2.01
RAL-IC number 4 vs. 5	0.055	2.39	0.98∼5.82
BGSF <0.9 vs. ≥0.9	0.04	2.01	1.01∼4.32
≥Grade 2 RC	0.000	2.47	1.65∼3.70

RC = rectal complication; BC = bladder complication; NRRII = non-rectal intestinal injury; RGSF = geometric sparing factor of the rectum; BGSF = geometric sparing factor of the bladder.

**TABLE 5:** Multivariate logistic regression estimated odds ratios (OR) for developing grade 2 or higher NRRII across different variables

**Variables**	***p* value**	**OR**	**95% CI**
Age < 65 vs. ≥65 years	0.29	0.46	0.11∼1.94
Age < 70 vs. ≥70 years	0.70	1.38	0.27∼7.10
Diabetes negative vs. positive	0.96	1.25	0.23∼8.95
Stage IB2-IIA vs. IIB-IIIB	0.47	1.20	0.72∼2.00
Stage IB2-IIB vs. IIIA-IIIB	0.34	1.45	0.68∼3.09
Non-IMRT vs. IMRT	0.33	0.64	0.26∼1.58
Parametrial dose ≤54 vs. > 54 Gy	0.31	0.63	0.25∼1.54
RAL-IC number 4 vs. 5	0.34	0.59	0.20∼1.76
RGSF < 0.7 vs. ≥0.7	0.42	2.14	0.33∼13.75
BGSF < 0.9 vs. ≥0.9	0.68	0.72	0.15∼3.41
≥Grade 2 RC	0.001	1.99	1.26∼3.16
≥Grade 2 BC	0.74	1.08	0.68∼1.73

RC = rectal complication; BC = bladder complication; NRRII = non-rectal intestinal injury; RGSF = geometric sparing factor of the rectum; BGSF = geometric sparing factor of the bladder.

**TABLE 6:** Patients with or without IMRT on having grade 2 or higher complications according to lower and higher 50% percentile of geometric sparing factor for rectum and bladder

**Groups**	**Complication**	**IMRT (%)**	**non-IMRT (%)**	*p*value*
Lower RGSF group (RGSF < 0.635) no = 156	RC (+)	1(2.2%)	6 (5.4%)	0.88
	RC (−)	43 (97.8%)	106 (94.6%)	
Higher RGSF group (RGSF > 0.635) no = 155	RC (+)	5 (13.2%)	16 (13.7%)	0.75
	RC (−)	33 (86.8%)	101 (86.3%)	
Lower BGSF group (BGSF < 0.695) no = 157	BC (+)	3 (7.0%)	17 (14.9%)	0.30
	BC (−)	40 (93.0%)	97 (85.1%)	
Higher BGSF group (BGSF > 0.695) no = 158	BC (+)	5 (12.5%)	18(15.3%)	0.97
	BC (−)	35 (87.5%)	100 (84.7%)	

Note:

* examined by Chi-square test

RC = rectal complication; BC = bladder complication; NRRII = non-rectal intestinal injury; RGSF = geometric sparing factor of the rectum; BGSF = geometric sparing factor of the bladder.
